# Study of heart rate recovery and cardiovascular autonomic modulation in healthy participants after submaximal exercise

**DOI:** 10.1038/s41598-021-83071-w

**Published:** 2021-02-11

**Authors:** Tábata P. Facioli, Stella V. Philbois, Ada C. Gastaldi, Daniel S. Almeida, Karina D. Maida, Jhennyfer A. L. Rodrigues, Juan C. Sánchez-Delgado, Hugo C. D. Souza

**Affiliations:** 1 Exercise Physiology Laboratory, Department of Biomechanics, Medicine and Rehabilitation, Ribeirão Preto Medical School, University of São Paulo, Av. Bandeirantes, 3900 - Vila Monte Alegre, Ribeirão Preto, SP 14049-900 Brazil; 2 Faculty of Health Sciences, Integral Physiotherapy Research Group, Universidad de Santander, Bucaramanga, Colombia

**Keywords:** Physiology, Cardiology, Hypertension

## Abstract

Heart rate variability (HRV), blood pressure variability (BPV), and baroreflex sensitivity (BRS) provide important information on cardiovascular autonomic control. However, little is known about the reorganization of HRV, BPV, and BRS after aerobic exercise. While there is a positive relationship between heart rate (HR) recovery rate and cardiorespiratory fitness, it is unclear whether there is a relationship between cardiorespiratory fitness and reorganization of cardiovascular autonomic modulation during recovery. Thus, this study aimed to investigate whether cardiorespiratory fitness influences the cardiovascular autonomic modulation recovery, after a cardiopulmonary exercise test. Sixty men were assigned into groups according to their cardiorespiratory fitness: low cardiorespiratory fitness (LCF = VO2: 22–38 mL kg^−1^ min^−1^), moderate (MCF = VO2: 38–48 mL kg^−1^ min^−1^), and high (HCF = VO2 > 48 mL kg^−1^ min^−1^). HRV (linear and non-linear analysis) and BPV (spectral analysis), and BRS (sequence method) were performed before and after a cardiopulmonary exercise test. The groups with higher cardiorespiratory fitness had lower baseline HR values and HR recovery time after the cardiopulmonary exercise test. On comparing rest and recovery periods, the spectral analysis of HRV showed a decrease in low-frequency (LF) oscillations in absolute units and high frequency (HF) in absolute and normalized units. It also showed increases in LF oscillations of blood pressure. Nonlinear analysis showed a reduction in approximate entropy (ApEn) and in Poincare Plot parameters (SD1 and SD2), accompanied by increases in detrended fluctuation analysis (DFA) parameters α1 and α2. However, we did not find differences in cardiovascular autonomic modulation parameters and BRS in relation to cardiorespiratory fitness neither before nor after the cardiopulmonary test. We concluded that cardiorespiratory fitness does not affect cardiovascular autonomic modulations after cardiopulmonary exercise test, unlike HR recovery.

## Introduction

The investigation of the influence of the autonomic nervous system through baroreflex sensitivity (BRS), heart rate variability (HRV), and blood pressure variability (BPV) provides important information on cardiovascular autonomic regulation integrity in the most varied conditions, especially in individuals with chronic and degenerative diseases, such as metabolic and cardiovascular diseases^1–3^. Thus, autonomic alterations characterized by a decrease in HRV and BRS as well as an increase in BPV, are important, since they might indicate greater risk of adverse cardiovascular events and sudden death^4^.

The autonomic assessment methodology, characterized mainly by HRV and BPV analysis, is relatively simple. It is based on measuring and recording heart rate (HR) and blood pressure (BP), with the individual at rest in supine position, and/or during autonomic provocation tests, such as the tilt test^1,5^. After obtaining HR and BP, the values can be analyzed by linear and non-linear analyses, with quite reliable results. It is possible that autonomic modulation evaluation of HRV and BPV as well as BRS, might be of importance after physical stress triggered by cardiopulmonary exercise testing, since other parameters obtained after physical exercise are used in clinical practice as references for cardiovascular health. One such parameter is the HR recovery rate. We know that individuals who present better cardiovascular performance during physical exercise in cardiopulmonary testing also present greater HR recovery, characterized by a greater decrease in HR in a shorter period. This recovery is a morbidity and mortality predictor in patients with cardiovascular diseases^6,7^.

However, few studies have investigated the cardiovascular autonomic function behavior during HR recovery, specifically, the capacity of autonomic reorganization after cardiopulmonary exercise testing. Thus, we are unaware regarding the relationship between cardiorespiratory fitness and autonomic cardiovascular control reorganization, mainly in autonomic modulation of HRV, BPV, and BRS. Therefore, we investigated whether cardiorespiratory fitness influences the recovery of cardiovascular autonomic modulation after a submaximal cardiopulmonary test.

## Methods

### Participants

Sixty healthy male participants, aged between 18 and 45 years, participated in this study. None of the participants were smokers, had any cardiovascular, musculoskeletal, or metabolic disorders, or any other disease that could compromise the performance and results of the tests. All participants were from the Exercise Physiology Laboratory, Ribeirão Preto Medical School, University of São Paulo, Brazil. They were informed about the legal and ethical aspects of the study and a written consent form was obtained, wherein they agreed to participate, prior to the start of the study. All study procedures were carried out in accordance with the guidelines stated the Declaration of Helsinki and Good Clinical Practice guidelines. This study was approved by the Ethics Committee on Human Research, Hospital das Clínicas de Ribeirão Preto, at the Medical School of the University of São Paulo (USP, SP, Brazil) (Protocol number: 045616/2014).

Prior to the experimental procedures, all participants were interviewed (anamnesis) to obtain information about health status and physical fitness (type, weekly frequency, and workload of exercise sessions). After anamnesis, the 60 selected participants were submitted to cardiopulmonary exercise testing and, according to maximum oxygen uptake (VO_2max_), subsequently assigned to one of three different groups (N = 20): low cardiorespiratory fitness (LCF, VO_2max_: 22–38 mL kg^−1^ min^−1^), moderate cardiorespiratory fitness (MCF, VO_2max_: 38–48 mL kg^−1^ min^−1^), and high cardiorespiratory fitness (HCF, VO_2max_: > 48 mL kg^−−1^ min^−1^). The divisions of the groups at different levels of O_2_ consumption were adapted according to the values established by the American Heart Association.

The maximal cardiopulmonary exercise test was performed using a treadmill (Super ATL Millenium, Inbramed/Inbrasport, Brazil) following the protocol described by Ellestad^8^, followed by a six-minute active recovery period with a speed of 2.5 km h^−1^ and an inclination of 2.5%. The analysis of exhaled gases (VO_2_ and VCO_2_) and other parameters was performed using a metabolic device (UltimaTM CardiO2, Medical Graphics Corp., St. Paul, MN, USA). The VO_2max_ was identified when the VO_2_ curve presented a plateau regardless of the increase in workload^9,10^.

During maximal cardiopulmonary exercise test, blood samples for analysis of lactate concentrations were collected from the ear lobe thrice: at rest (baseline), in VO_2max_, and 10 min after recovery initiation. Disposable lancets and calibrated heparinized capillary tubes were used. Blood was stored in Eppendorf tubes (1.5 mL) containing 50 μL NaF (1% sodium fluoride) and then analyzed by an enzymatic method.

### Study design

The participants were invited to attend two laboratory visits, both in the morning, between 09:00 and 11:00 am, with a 48-h interval between visits. During the first visit, anthropometric data were collected, and the maximal cardiopulmonary test was performed, as described above, to assign the participants into groups according to the obtained VO_2max_.

During the second visit, baseline HR and BP were recorded for 20 min with participants in the supine position and the records were used for further analysis of HRV, BPV, and spontaneous BRS. The HR records were obtained using the RR intervals (RRi) from the electrocardiographic recording (Dual Bio Amp/Stimulator, ADInstruments, Australia), using a modified CM5 shunt at a sampling frequency of 1000 Hz. The BP records were obtained from digital plethysmography recording equipment (Finometer Pro, Finapres Medical System, Amsterdam, Netherlands), using a cuff positioned on the middle finger of the right upper limb. The data interface to the microcomputer was performed using the PowerLab4/35 device (ADInstruments, Australia). The data were recorded and stored (Software LabChart 7.0, ADInstruments, Australia) for further analysis.

Afterwards, the devices of the Finometer equipment were removed from the participants and cardiopulmonary submaximal test was performed, establishing the intensity between 90 and 95% of the maximum HR obtained in the maximum cardiopulmonary test performed on the first visit^8^. After the target HR had been reached, the recovery period had the following sequence, lasting 36 min; the volunteers performed an active recovery lasting 6 min at a speed of 3 km h^−1^ and an inclination of 2.5°. After active recovery, the volunteers sat on a chair for 10 min to remove the mask from the gas analyzer and to replace the Finometer device to record pulsatile BP in addition to the HR (electrocardiography [ECG]) that was already being monitored since the beginning of the experimental procedure. After placing the Finometer devices, that is, in the 16th min of the recovery period, the volunteers were placed in a supine position on a stretcher, and the Finometer was calibrated again. Then, a 20-min record was documented. However, only the final 10 min (from the 26th to the 36th min) of the ECG and pulsatile BP records were used for HRV, BPV, and BRS analyses.

The participants had been instructed to not perform intense physical activity and avoid the consumption of alcoholic and caffeinated beverages for 48 h prior to the test. In addition, they were advised to sleep for at least 7–8 h, the night before the test. The room temperature was maintained at 21 °C, and the ambient light and noise was controlled to prevent any interference with data recording.

### Hemodynamic parameters

During the resting period, in the supine position as well as during the recovery period, systolic blood pressure (SBP), diastolic blood pressure (DBP), and mean blood pressure (MBP) were obtained using Finometer equipment. HR was obtained using an electrocardiographic digital recorder throughout the protocol, including cardiopulmonary testing (ML866 PowerLab, ADInstruments, Bella Vista, Australia).

### Anthropometric parameters

Body weight and height were obtained using an analog scale with an altimeter (Welmy), while the body mass index (BMI) values were obtained using the formula W/H^2^, where W is weight in kilograms and H is the height in meters of the participant. Body composition was determined using the bioelectrical impedance method (Quantum BIA 101; Q-RJL Systems, Clinton Township, MI, USA).

### Linear analysis—heart rate variability and blood pressure variability

HR records were obtained using the RRi from the ECG recordings. BP records for variability analysis were obtained from the beat-to-beat SBP by means of digital plethysmography recording equipment (Finometer).

As previously described, ECG and pulsatile BP were recorded during two different periods, before and after the submaximal cardiopulmonary test. In both moments, the participants were instructed to remain in the supine position for approximately 10 min to stabilize cardiovascular parameters. After this period, the ECG and BP pulsatile were recorded simultaneously for another 10 min. The BP plethysmographic recording equipment (Finometer) was calibrated before each data collection, using PhysioCal (physiological calibration) and return-to-flow (RTF), in addition to the photoplethysmographic height sensor. This procedure allowed the adjustment of peripheral pressure values (cuff on the middle finger) compared to brachial artery pressure values (cuff positioned in the ipsilateral upper region arm)^11^. The BPV and HRV analyses were performed using custom computer software (CardioSeries v2.0, http://sites.google.com/site/cardioseries) developed by Dias, DPM of the University of São Paulo, Brazil^12,13^. The RRi and SBP values were redesigned in 3 Hz cubic spline interpolation to normalize the time interval between the beats. The stationary segment was visually inspected, and those with artifacts or transients were excluded. Each RRi and SBP stationary segment was subjected to spectral analysis by fast Fourier transform (FFT), after Hanning window^14^. The RRi time series were integrated in bands of low frequency (LF; 0.04–0.15 Hz) and high frequency (HF; 0.15–0.5 Hz), and the results are expressed in absolute values (ms^2^) and normalized units (nu), while the SBP time series were integrated only in the low-frequency band (LF; 0.04–0.15 Hz), and the results are expressed in absolute values (mmHg^2^).

The HRV normalized values were obtained by calculating the percentage of LF and HF power related to the total power of the spectrum minus the very low-frequency band (VLF; < 0.2 Hz)^15^. In addition, the normalization procedure was performed to minimize variations in total power in LF and HF absolute values^1,16^. To assess sympathovagal balance, the LF/HF ratio of RRi variability was also calculated^17^.

### Non-linear analysis of HRV

RRi records were analyzed using the Kubios HRV Standard software (University of Eastern Finland, Kuopio, Finland)^18^. The recording artifacts were excluded by eliminating RRi that differed by more than 25% from previous and subsequent RRi and were replaced by conventional spline interpolation following the methodology described in previous studies and applying the media filter provided by Kubios HRV Standard^19^. The previous smoothness approach with a Lambda value of 500 was used to remove invalid low-frequency baseline trend components^18^.

### Spontaneous Baroreflex sensitivity

BRS was assessed in the time domain using the sequence technique, as described by Silva et al.^20^, also during two moments, before and after the submaximal cardiopulmonary test. The computer software CardioSeries v2.1 scanned beat-to-beat time series of pulse interval (PI) and SBP values, searching for sequences of at least four consecutive beats, in which progressive increases in SBP were followed by progressive increases in PI (up sequence) and progressive decreases in SBP were followed by progressive decreases in PI (down sequence), with a correlation coefficient (r) between PI and SBP values higher than 0.8. Spontaneous BRS was determined by the linear regression line to the mean slope between the SBP and PI values of each sequence found. The number of baroreflex sequences found (per 1000 beats) and the mean individual significant SBP/PI slope relationship, obtained by averaging all slopes computed within the test period, was calculated and taken as a measure of spontaneous BRS.

### Statistical analysis

The results are presented as mean ± standard deviation (SD). The effects of cardiorespiratory fitness on anthropometric parameters, VO_2max_, blood pressure, lactate concentrations and heart rate were assessed by one-way analysis of variance (ANOVA). When appropriate, post hoc comparisons were performed using the Tukey test. In turn, the effects of the level of cardiorespiratory fitness on HRV, blood pressure variability, and BRS were assessed by two-way ANOVA. When appropriate, post hoc comparisons were performed using the Student–Newman–Keuls method. Differences were considered significant when p < 0.05. All statistical tests were performed using SigmaPlot 11.0 software (Systat Software Inc., San Jose, CA, USA). The “GraphPad StatMate 2.0” did the sample size calculation, confidence level was set at 95%, power of 80%, with the LF and HF variables in normalized units. The sample size was set to 20 participants per group.

## Results

The anthropometric characteristics and hemodynamic parameter values of the participants are shown in Table 1. The HCF group presented lower weight, BMI, and lower body fat percentage compared to the other groups. In addition, those who performed better in the cardiopulmonary test had higher VO_2max_ values.

**Table 1:** Anthropomorphic characteristics and hemodynamic parameters of all groups.

	Low	Moderate	High	*P*
**Characteristics**				
Age, years	29 ± 7.1	31 ± 6.8	33 ± 6.3	0.2
Height, m	1.77 ± 0.05	1.76 ± 0.06	1.77 ± 0.06	0.57
Weight, kg	80 ± 8.5	80 ± 12	70 ± 10^a,b^	< 0.001
BMI, kg m^−2^	25.5 ± 2.0	25.8 ± 3.4	22.4 ± 2.4^a,b^	< 0.001
% Body fat	16.4 ± 3.7	16.7 ± 3.4	10.7 ± 1.7^a,b^	< 0.001
VO_2max_, mL kg^−1^ min^−1^	37.6 ± 2.5	46.3 ± 2.4 ^*a*^	66.3 ± 9.6^a,b^	< 0.001
**Hemodynamic parameters**				
SBP, mmHg	121 ± 12	121 ± 13	115 ± 11	0.57
DBP, mmHg	79 ± 8.6	75 ± 11	74 ± 6.2	0.06
MBP, mmHg	93 ± 7.8	91 ± 10	88 ± 6.7	0.13

Values are presented as mean ± SD.

*m* meter, *kg* kilograms, *%* percentage, *VO*_*2max*_ oxygen consumption maximum, *mL* milliliters, *min* minute, *mmHg* millimeters of mercury, *SBP* systolic blood pressure, *DBP* diastolic blood pressure, *MBP* mean blood pressure.

^a^P < 0.05 *vs.* low cardiorespiratory fitness group.

^b^P < 0.05 *vs.* moderate cardiorespiratory fitness group.

On the contrary, there were no differences in SBP, DBP, and MBP values among the different levels of cardiorespiratory fitness (Table 1).

Table 2 shows the HR values during rest, peak exercise, and recovery (active and supine position). It also shows the HR variation delta (∆) during recovery. The HCF group had lower HR values at rest when compared to that in other groups. Peak HR was similar across all three groups. On the contrary, during recovery, the HCF group had lower values of HR and ∆HR, when compared to that in other groups, indicating a better recovery HR.

**Table 2:** Values of heart rate and heart rate variation delta (∆) obtained in all groups at rest, at peak of cardiopulmonary test and during recovery.

	Low	Moderate	High	*P*
**Heart rate**				
HR_resting_, bpm	78 ± 12	69 ± 10^a^	55 ± 4^a,b^	< 0.001
HR_peak_, bpm	181 ± 12	181 ± 8	181 ± 9	0.975
**Heart rate recovery**				
HR_1min after peak_, bpm	163 ± 14	155 ± 10^a^	148 ± 12^a,b^	0.001
HR_3min after peak_, bpm	141 ± 16	131 ± 16	122 ± 14^a^	0.001
HR_6min after peak_, bpm	128 ± 15	121 ± 15	111 ± 10^a,b^	< 0.001
HR_16min after peak_, bpm	91 ± 12	90 ± 11	84 ± 9	0.118
HR_26min after peak_, bpm	89 ± 12	88 ± 11	83 ± 9	0.117
HR_36min after peak_, bpm	83 ± 10	79 ± 9	72 ± 7^a,b^	< 0.001
∆** heart rate recovery**				
∆HR_1min after HR peak_, bpm	− 18 ± 6.3	− 26 ± 5.6^a^	− 32 ± 11^a,b^	< 0.001
∆HR_3min after HR peak_, bpm	− 40 ± 11	− 49 ± 14^a^	− 58 ± 12^a,b^	< 0.001
∆HR_6min after HR peak_, bpm	− 52 ± 10	− 60 ± 12^a^	− 69 ± 9^a,b^	< 0.001
∆HR_16min after HR peak_, bpm	− 90 ± 11	− 91 ± 9	− 96 ± 10	0.149
∆HR_26min after HR peak_, bpm	− 91 ± 12	− 93 ± 9	− 98 ± 11	0.141
∆HR_36min after HR peak_, bpm	− 96 ± 9	− 101 ± 7	− 108 ± 6^a,b^	< 0.001

Values are presented as mean ± SD.

*HR* heart rate; bpm, beats per minute.

^a^P < 0.05 vs. low cardiorespiratory fitness group.

^b^P < 0.05 vs. moderate cardiorespiratory fitness group.

Table 3 present the results of linear (spectral analysis) and non-linear analysis of HRV, both during baseline recording (supine rest) and recovery (supine recovery). Comparing rest and recovery period, the spectral analysis showed decrease in variance, LF in absolute units and HF in absolute and normalized units. It also showed increases in LF oscillations in normalized units and in the LF/HF ratio. Nonlinear analysis showed a reduction in entropy (ApEn) and in Poincare Plot parameters (SD1 and SD2), accompanied by increases in DFA parameters, α1 e α2.

**Table 3:** Volunteer’s cardiac autonomic parameters evaluated at supine positions before and after cardiopulmonary test.

	Rest supine			Recovery supine			Cardiorespiratory Fitness factor		recovery factor		Interaction	
	Low	Moderate	High	Low	Moderate	High	F_(DF)_	*P*	F_(DF)_	*P*	F_(DF)_	*P*
RRi	929 ± 144	925 ± 102	1065 ± 144	660 ± 87	678 ± 91	712 ± 69	F_(2,114)_: 9.1	< 0.001	F_(1,114)_: 208	< 0.001	F_(2,114)_: 2.6	0.08
**Linear analysis**												
Variance, ms^2^	2317 ± 1559	2382 ± 1114	2440 ± 2159	386 ± 405	316 ± 252	408 ± 311	F_(2,114)_: 0.05	0.95	F_(1,114)_: 84	< 0.001	F_(2,114)_: 0.03	0.97
LF, ms^2^	894 ± 835	949 ± 723	839 ± 856	183 ± 226	121 ± 121	159 ± 115	F_(2,114)_: 0.1	0.94	F_(1,114)_: 48	< 0.001	F_(2,114)_: 0.2	0.83
LF, nu	60 ± 22	62 ± 15	54 ± 20	81 ± 13	83 ± 6.8	77 ± 22	F_(2,114)_: 1.8	0.16	F_(1,114)_: 47	< 0.001	F_(2,114)_: 0.04	0.96
HF, ms^2^	701 ± 796	521 ± 319	725 ± 719	40 ± 58	20 ± 14	46 ± 41	F_(2,114)_: 0.8	0.48	F_(1,114)_: 54	< 0.001	F_(2,114)_: 0.46	0.63
HF, nu	40 ± 22	38 ± 15	46 ± 20	18 ± 13	17 ± 6.8	23 ± 22	F_(2,114)_: 1.8	0.16	F_(1,114)_: 47	< 0.001	F_(2,114)_: 0.04	0.96
LF/HF ratio	2.6 ± 3.2	2.0 ± 1.2	1.8 ± 1.6	8.2 ± 6.6	6.7 ± 6.4	6.6 ± 5.7	F_(2,114)_: 0.7	0.48	F_(1,114)_: 35	< 0.001	F_(2,114)_:0.1	0.89
**Non-linear analysis**												
ApEn	1.32 ± 0.11	1.34 ± 0.14	1.27 ± 0.11	1.15 ± 0.21	1.21 ± 0.17	1.23 ± 0.17	F_(2,114)_: 0.7	0.51	F_(1,114)_:16.2	< 0.001	F_(2,114)_: 2.2	0.12
DFA-α1	1.07 ± 0.29	1.09 ± 0.17	0.99 ± 0.28	1.56 ± 0.21	1.55 ± 0.11	1.53 ± 0.22	F_(2,114)_: 0.8	0.43	F_(1,114)_: 146	< 0.001	F_(2,114)_: 0.5	0.64
DFA-α2	0.41 ± 0.15	0.42 ± 0.11	0.38 ± 0.13	0.64 ± 0.18	0.65 ± 0.18	0.54 ± 0.14	F_(2,114)_: 2.7	0.09	F_(1,114)_: 58	< 0.001	F_(2,114)_: 0.8	0.44
SD1, ms	29 ± 15	31 ± 18	38 ± 20	8 ± 7	5 ± 3	9 ± 7	F_(2,114)_: 1.1	0.34	F_(1,114)_: 76	< 0.001	F_(2,114)_: 0.2	0.81
SD2, ms	54 ± 24	59 ± 22	55 ± 27	23 ± 21	18 ± 11	23 ± 11	F_(2,114)_: 0.02	0.99	F_(1,114)_: 86	< 0.001	F_(2,114)_: 0.5	0.63

Data are presented as mean ± SD.

*RRi* R–R intervals of ECG, *ms* milliseconds, *LF* low-frequency band, *HF* high frequency band, *nu* normalized units, *ApEn* approximate entropy, *DFA* detrended fluctuation analysis, *SD1* standard deviation of instantaneous beat-to-beat interval variability, *SD2* standard deviation of continuous long-term R–R interval variability, *F* factor, *DF* degrees of freedom.

Table 4 present the results of BPV and BRS analysis, both obtained during baseline recording (supine rest) and recovery (supine recovery). Comparing rest and recovery period, the SBP analysis showed an increase of LF oscillations. In turn, the BRS parameters were reduced. In contrast, there was no cardiorespiratory fitness influence on HRV and BPV parameters and BRS (Tables 3 and 4).

**Table 4:** Volunteer’s cardiovascular autonomic parameters evaluated at rest and supine positions.

	Rest supine			Recovery supine			Cardiorespiratory fitness factor		Recovery factor		Interaction	
	Low	Moderate	High	Low	Moderate	High	F_(DF)_	*P*	F_(DF)_	*P*	F_(DF)_	*P*
**BP variability**												
Variance, mmHg^2^	19.1 ± 10.7	26.2 ± 13.9	19.5 ± 10.9	21.4 ± 11.5	25.2 ± 12.6	23.3 ± 13.0	F_(2,114)_: 2.2	0.110	F_(1,114)_:0.5	0.459	F_(2,114)_: 0.4	0.660
LF, mmHg^2^	6.3 ± 4.4	9.3 ± 5.6	5.6 ± 3.7	9.2 ± 5.1	10.4 ± 8.5	10.2 ± 5.5	F_(2,114)_: 1.7	0.177	F_(1,114)_: 7.8	0.006	F_(2,114)_: 0.9	0.373
**Spontaneous BRS**												
BEI	0.64 ± 0.15	0.7 ± 0.15	0.57 ± 0.18	0.27 ± 0.21	0.28 ± 0.22	0.31 ± 0.18	F_(2,114)_: 0.7	0.485	F_(1,114)_: 101	< 0.001	F_(2,114)_: 1.8	0.161
Ramps nº	83.9 ± 42	78.7 ± 29	50.8 ± 21	41.2 ± 27	42.6 ± 25	40.4 ± 24	F_(2,114)_:8,3	< 0.001	F_(1,114)_: 22,5	< 0.001	F_(2,114)_: 2.2	0.116
Gain, ms mmHg^−1^	14.4 ± 10.8	12.3 ± 4.8	18.2 ± 10.5	4.3 ± 3.1	3.6 ± 1.6	4.9 ± 2.7	F_(2,114)_: 2.8	0.061	F_(1,114)_: 75	< 0.001	F_(2,114)_: 1.2	0.308
Gain down, ms mmHg^−1^	14.7 ± 10.6	12.4 ± 4.5	18.4 ± 10.6	4.5 ± 3.4	3.7 ± 1.6	5.5 ± 3.0	F_(2,114)_: 3.3	0.060	F_(1,114)_: 72	< 0.001	F_(2,114)_: 0.97	0.382
Gain up, ms mmHg^−1^	14.1 ± 11.2	12.4 ± 5.3	17.8 ± 11.5	3.7 ± 1.9	3.5 ± 1.6	4.6 ± 2.2	F_(2,114)_: 2.2	0.111	F_(1,114)_: 70	< 0.001	F_(2,114)_: 0.9	0.400

Data are presented as mean ± SD.

*BP* blood pressure, *mmHg* millimeter of mercury, *LF* low frequency, *BRS* baroreflex sensitivity, *BEI* baroreflex effectiveness index, *ms/mmHg* milliseconds/millimeter of mercury, *gains up* increase in the pulse interval (bradycardia) resulting from increased blood pressure, *gain down* reduction in the pulse interval (tachycardia) resulting from a reduction in blood pressure, *F* factor, *DF* degrees of freedom.

**Figure 1:**
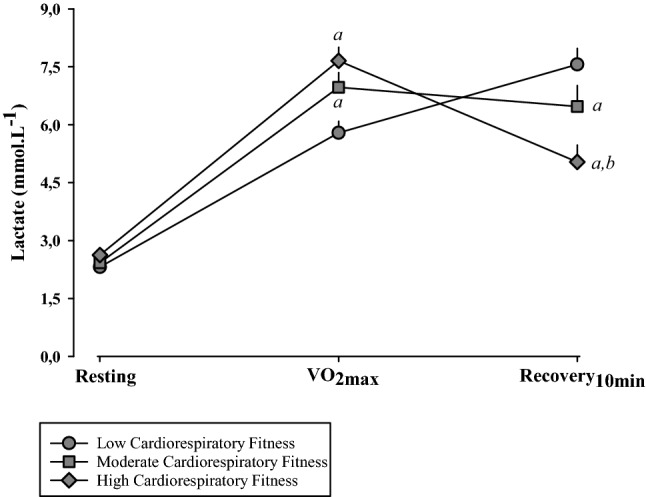
Peripheral blood lactate obtained during the maximal cardiopulmonary test at three different moments: resting; VO_2max_; and 10 min after the start of recovery (Recovery 10 min); mmol L^−1^, millimol per liter; ^a^P < 0.05 vs. Low Cardiorespiratory Fitness group; ^b^P < 0.05 vs. Moderate Cardiorespiratory Fitness group.

Figure 1 and Table 5 shows the blood lactate values during rest (resting), maximal cardiopulmonary test plateau (VO_2max_), and 10 min after the cardiopulmonary test maximum plateau (recovery period—10 min). During VO_2max_, lactate concentrations were higher in HCF and MCF groups, when compared to LCF. However, during recovery (10 min), the HCF group showed higher lactate uptake, characterized by a lower concentration of this substrate, when compared to that in other groups.

**Table 5:** Peripheral blood lactate obtained during the maximal cardiopulmonary test (VO_2max_) at three different moments: resting; VO_2max_; and 10 min after the start of recovery (Recovery 10_ min_).

	Resting	VO_2max_	Recovery 10_ min_	*P*
**Lactate, mmol L**^**−1**^				
Low fitness	2.3 ± 0.8	5.8 ± 1.3^a^	7.6 ± 1.9^a,b^	< 0.001
Moderate fitness	2.4 ± 0.7	6.9 ± 1.7^a^	6.5 ± 2.4^a^	< 0.001
High fitness	2.6 ± 0.4	7.7 ± 1.6^a^	5.0 ± 2.0^a,b^	< 0.001

Values are presented as mean ± SD.

mmol L^−1^ millimol per liter.

^a^P < 0.05 *vs.* resting.

^b^P < 0.05 *vs.* VO_2max_.

## Discussion

The present study investigated the reorganization of cardiovascular autonomic modulation and baroreflex sensitivity after submaximal aerobic exercise. Our results showed that, contrary to what happens with heart rate recovery, cardiorespiratory fitness does not seem to influence the reorganization of the autonomic parameters evaluated, at least in the short term.

The cardiorespiratory fitness relationship with different physiological parameters is known. Often, we observe cardiac, metabolic and hormonal morphophysiological adaptations induced by physical training, mainly by aerobic exercise. Among cardiac morphophysiological adaptations, eccentric cardiac hypertrophy associated with increased diameter and final diastolic volume is frequently observed, resulting in a significant increase in ejection volume. These adaptations are directly related to lower baseline HR and higher HR rate decline, during the recovery period. This HR rate recovery has been widely used as an important index of cardiovascular morbidity and mortality^6,7^.

Our results corroborate the literature as they show a negative relation between cardiorespiratory fitness and HR values at rest. The higher the cardiorespiratory fitness, the lower the rest HR values. Likewise, a higher HR recovery rate was also observed, after the cardiopulmonary exercise test. According to the literature, the marked reduction in HR, immediately after exercise, might be related to a decrease in cardiac output by intrinsic self-regulation. The ejection volume remains higher in trained individuals due to redirection of peripheral blood to central regions, which increases venous return and facilitates ventricular filling^21,22^. It has also been suggested that physical training might promote alterations in cardiac autonomic tonic balance, characterized by an autonomic balance more favorable to vagal component activity^6,23^.

Thus, it is likely that individuals with greater cardiorespiratory fitness present a different cardiovascular autonomic activity balance, when compared to individuals with lower cardiorespiratory fitness. However, a higher vagal activity is different from a higher vagal cardiac autonomic modulation, and the same observation applies to the sympathetic component. In this case, although the possible changes in cardiovascular autonomic activity due to greater cardiorespiratory fitness, which were not investigated, might results in similar autonomic modulation, as observed in the present study and in previous studies^12,24^. Thus, cardiorespiratory fitness does not seem to influence the reorganization of cardiac autonomic modulation, corroborating a previous study^23^. In this case, the linear and non-linear assessment showed coherence, since there were also no differences in the non-linear parameters assessed in relation to cardiorespiratory fitness, neither before nor after the cardiopulmonary test. The HRV and HR recovery discrepancies might be attributed to differences between them; HRV reflects a phasic control of vagal influence, while HR recovery reflects the mean cholinergic (tonic control) of the sinoatrial node, a tonic effect^25^. Thus, it is possible that during the recovery period, after the submaximal cardiopulmonary test, sympathetic activity is decreasing and vagal activity is increasing, while modulation of HRV and BPV do not represent same results^1,26^. This fact seems to be a contradictory response, however, as pointed above, activity and autonomic modulation are different properties. Another hypothesis involves mechanism of to attenuate the activation of the excitatory parasympathetic and inhibitory sympathetic efferent pathway, during physical exercise, which lasts for a while, after the exercise ends^27^, in this case the 20 min recovery period, analyzed in the present study, might not have been enough to HRV reestablishment, even when applied in groups with different cardiorespiratory fitness. Studies have shown that HRV can remain decreased within 1 h after heavy exercise^28,29^. However, the cause is uncertain, it seems that recovery from cardiovascular autonomic modulation after exercise is not affected by cardiorespiratory fitness, as observed during rest^12,24^.

There were no differences between our groups in BPV and BRS during recovery. However, the BPV-LF component was increased during recovery, when compared to rest, suggesting an increment of vascular sympathetic autonomic modulation. It is known that decreased BRS might indicate that the role of baroreflex in acute recovery of BP, after a submaximal exercise, is not efficient and perhaps it is less important, due to high systemic cardiovascular demand^30^.

During recovery, despite the removal of circulating metabolites and catecholamines, blood pH and body temperature reestablishment^31,32^, there is still a decrease in action of chemoreceptors, thermoreceptors and muscle metaboreceptors^32–34^. It is known that metaboreceptors are associated with the baroreflex adjustment that occurs during physical exercise^34,35^. Thus, it seems that the role played by metaboreceptors is more important than the baroreflex, during recovery^35^. In this sense, increased levels of lactate, observed during recovery in all groups studied, even the less pronounced in the HCF group, due to better cardiorespiratory fitness^36,37^, corroborates studies showing that high levels of lactate, after exercise, might be responsible for spontaneous BRS attenuation, since they may allow for a greater action of metaboreceptors^30,35^. Therefore, the high production of metabolites, associated with persistent sympathetic modulation observed in the present study, could explain at least in part, the delay in the basal autonomic modulation return. It is important to highlight that the increase in body temperature generated by physical exercise stimulates skin receptors and generates a sympathetic response for the heart, which requires a decrease in metabolic activity for cooling and consequent sympathetic reduction^38^. Since baroreflex recovery is slower after intense exercises^31,39^, the mechanisms likely involved in decreasing the baroreflex response during exercise, such as the GABAergic neurons and the paraventricular nucleus (PVN) vasopressinergic neurons, would continue to promote a minor response from the baroreflex system^27^, and would be reestablished over time. However, the HRV and BPV recording period, in the present study, might not have been enough to promote a BRS restoration^40^.

In fact, the literature data scarcity about autonomic modulation response during recovery after exercise in individuals with different levels of physical conditioning, coupled to methodological differences employed, prevent to fully understand the autonomic modulation regulation^28,29^.

However, our results suggest that cardiorespiratory fitness has no short-term effect on HRV, BPV and BRS reorganization in healthy young and middle age mens after a submaximal exercise test. We hypothesize that cardiovascular autonomic control is already operating properly in healthy individuals, independently of their cardiorespiratory fitness, thus physical training would have no additive effect.

Thus, level of physical conditioning, in general, does not influence the cardiovascular parameters analyzed in this study, during recovery after submaximal exercise.

## Study limitations

The main limitation of the study was that HRV, BPV and SBR were not evaluated over a longer period. It is possible that a longer time may have differences between levels of cardiorespiratory fitness. Another important aspect is the need to include other forms of analysis. We are working on having these questions addressed and answered. It is important to note that the study limitations do not invalidate the main findings and their clinical implications. In addition to addressing the limitations of the study in future investigations, we seek to expand knowledge of the regulation mechanisms of cardiovascular autonomic control, as well as the effect of physical fitness on these mechanisms.
